# Can Speaker Gaze Modulate Syntactic Structuring and Thematic Role Assignment during Spoken Sentence Comprehension?

**DOI:** 10.3389/fpsyg.2012.00538

**Published:** 2012-12-05

**Authors:** Pia Knoeferle, Helene Kreysa

**Affiliations:** ^1^Cognitive Interaction Technology Excellence Center, Bielefeld UniversityBielefeld, Germany

**Keywords:** visually situated sentence comprehension, speaker gaze, visual context effects, sentence structure, eye tracking

## Abstract

During comprehension, a listener can rapidly follow a frontally seated speaker’s gaze to an object before its mention, a behavior which can shorten latencies in speeded sentence verification. However, the robustness of gaze-following, its interaction with core comprehension processes such as syntactic structuring, and the persistence of its effects are unclear. In two “visual-world” eye-tracking experiments participants watched a video of a speaker, seated at an angle, describing transitive (non-depicted) actions between two of three Second Life characters on a computer screen. Sentences were in German and had either subject*_NP1_*-verb-object*_NP2_* or object*_NP1_*-verb-subject*_NP2_* structure; the speaker either shifted gaze to the NP2 character or was obscured. Several seconds later, participants verified either the sentence referents or their role relations. When participants had seen the speaker’s gaze shift, they anticipated the NP2 character before its mention and earlier than when the speaker was obscured. This effect was more pronounced for SVO than OVS sentences in both tasks. Interactions of speaker gaze and sentence structure were more pervasive in role-relations verification: participants verified the role relations faster for SVO than OVS sentences, and faster when they had seen the speaker shift gaze than when the speaker was obscured. When sentence and template role-relations matched, gaze-following even eliminated the SVO-OVS response-time differences. Thus, gaze-following is robust even when the speaker is seated at an angle to the listener; it varies depending on the syntactic structure and thematic role relations conveyed by a sentence; and its effects can extend to delayed post-sentence comprehension processes. These results suggest that speaker gaze effects contribute pervasively to visual attention and comprehension processes and should thus be accommodated by accounts of situated language comprehension.

## Introduction

Past research has provided ample evidence that information in the non-linguistic context can incrementally modulate a listener’s visual attention during real-time sentence comprehension. This has been shown for aspects of the visual context such as size contrast between objects (Sedivy et al., 1999), their shape (Dahan and Tanenhaus, 2005), the semantic relationships between objects (Huettig and Altmann, 2004), referential contrast (Tanenhaus et al., 1995), depicted clipart events (Knoeferle et al., 2005), real-world action events (Knoeferle et al., 2011), action affordances (Chambers et al., 2004), the spatial location of objects (Altmann, 2004), gestures (e.g., Campana et al., 2005), and the speaker’s locus of gaze (e.g., Hanna and Brennan, 2008).

To accommodate these effects, accounts of language comprehension (e.g., the Coordinated Interplay Account, CIA; Knoeferle and Crocker, 2007) assume that words in the utterance guide (visual) attention to relevant aspects of the visual context or their mental representation; the words are co-indexed with the attended scene information, and the latter can then influence language comprehension and visual attention. However, the existing processing accounts (see also, e.g., Altmann and Kamide, 2007, 2009) do not yet accommodate the behavior of the speaker him/herself, despite the fact that speaker-based information such as iconic gestures (Wu and Coulson, 2005), beat gestures (Holle et al., 2012), and a speaker’s gaze can rapidly affect language comprehension.

For instance, in an eye-tracking study on effects of speaker gaze, a speaker and a listener faced each other with two arrays of shapes between them (Hanna and Brennan, 2008). A typical trial consisted of the speaker first inspecting and then mentioning one of two blue circles (target: with five dots; competitor: with a different number of dots). Approximately 1000 ms after the listener had heard *blue* in *blue circle* and before the utterance disambiguated the target by specifying the number of dots (*with five dots*), the listener looked more at the target than at the competitor, suggesting that speaker gaze disambiguated a referentially ambiguous target object (Hanna and Brennan, 2008; Experiment 1). In a related experiment, a robot that faced the participant frontally described size relations between objects in the scene; the description was either true or false, and the robot either looked toward the object it was about to mention, or it looked at an object other than the one it would mention, or it looked straight ahead and thus at none of the objects. Participants were highly likely to follow a robot speaker’s gaze shift (Staudte and Crocker, 2011; Experiment 1).

To sum up, speaker gaze has been shown to permit the anticipation of upcoming referents in settings in which the speaker faced the listener fully frontally (Hanna and Brennan, 2008; Staudte and Crocker, 2011). In addition, participants who did (vs. did not) follow the robot’s gaze showed larger gaze congruence effects in their sentence verification times (shorter response latencies for congruent vs. incongruent robot gaze, Staudte and Crocker, 2011; see also Richardson and Dale (2005), for reports that coordination of speaker and listener gaze can improve listeners’ performance on comprehension questions compared to a randomized baseline).

The present research aims to extend the existing findings in several regards. First, we asked whether gaze effects are robust even when the speaker does not face the listener fully frontally. Although the precision of gaze-direction detection is high when facing another person, it decreases as that person turns sideways (e.g., Gibson and Pick, 1963; Cline, 1967). Thus, speaker gaze might affect a listener’s visual attention rapidly only when the listener can see both of the speaker’s eyes, and when head movements can be detected easily. Alternatively, this may be possible even when she is positioned at an angle (e.g., at 45–60°, see Table 1), a finding that would underscore the robustness of speaker gaze effects.

**Table 1:** **Overview of the experimental conditions (congruence is not depicted)**.

Condition	Gaze	Sentence structure	Video	Sentence
(a)	Gaze	SVO	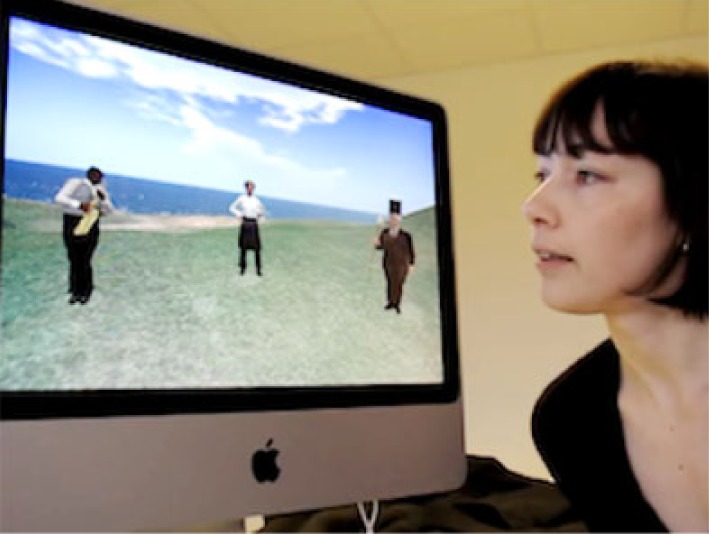	*Der Kellner beglückwünscht den Millionär außerhalb des Geschäfts*
(b)	Gaze	OVS	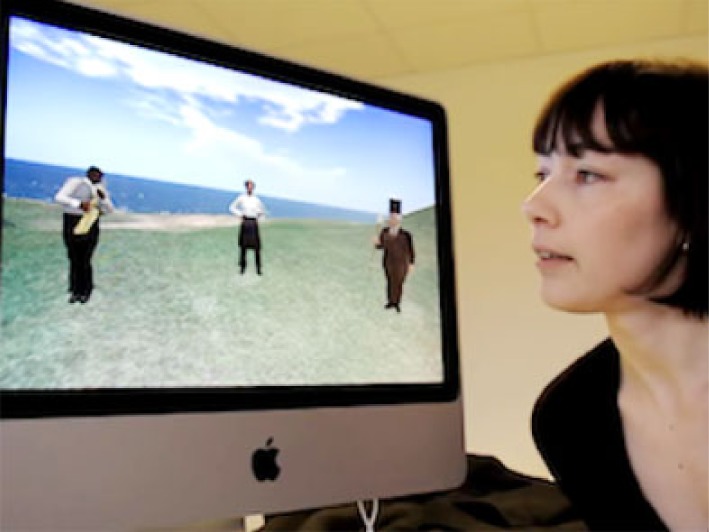	*Den Kellner beglückwünscht der Saxofonist außerhalb des Geschäfts*
(c)	No gaze	SVO	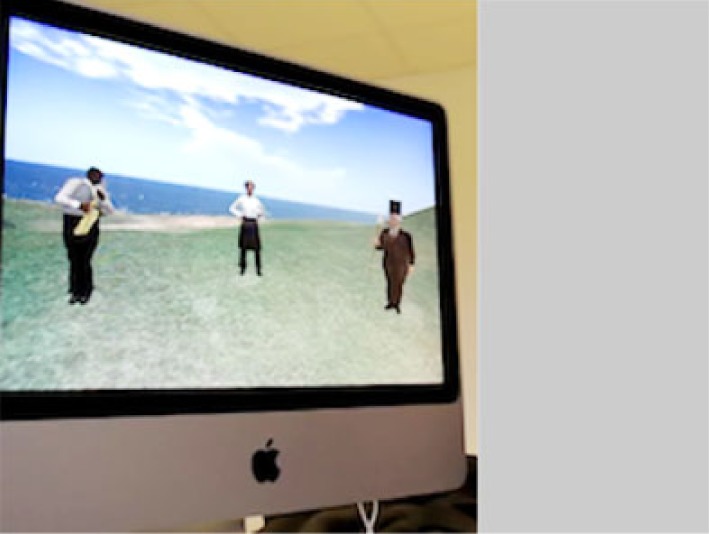	*Der Kellner beglückwünscht den Millionär außerhalb des Geschäfts*
(d)	No gaze	OVS	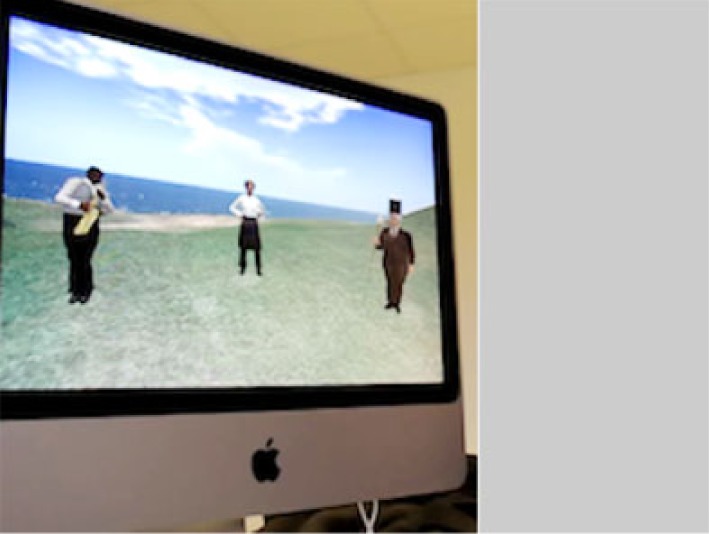	*Den Kellner beglückwünscht der Saxofonist außerhalb des Geschäfts*

*The English translation of the SVO sentence is “the waiter congratulates the millionaire outside the shop,” while the OVS sentence implies that the waiter is *being* congratulated by the saxophone player. The video is represented by a snapshot illustrating the gaze condition*.

Second, we asked whether speaker gaze can affect processes such as syntactic structuring and thematic role assignment in addition to referential anticipation. If the speaker’s gaze – much like action and object information – interacts with syntactic structure building and thematic role assignment, we should see differential effects on the processing of sentence structures that vary in the canonicality of the grammatical and thematic role relations they convey (see, e.g., Tanenhaus et al., 1995; Chambers et al., 2004; Knoeferle et al., 2005). Alternatively, speaker gaze-based referent anticipation occurs “across the board,” in which case its time course should be similar for different sentence structures.

Finally, we know little about the temporal persistence of speaker gaze effects. Staudte and Crocker (2011) reported that gaze-following during comprehension (vs. the failure to follow the speaker’s gaze) led to faster response times in a sentence verification task. The temporal persistence of such gaze-following effects, however, is not clear from their results, since participants on average responded immediately at sentence end.

Two “visual-world” eye-tracking experiments examined these three open issues. Participants inspected videos in which the thematic role relations between two out of three virtual characters, displayed on a computer screen, were described either by a visually present speaker or by a disembodied voice (the speaker was grayed out by a superimposed bar). The speaker’s eyes were only partially visible, since she was videotaped at a 45–60° angle relative to the camera. Sentences had either subject-verb-object (SVO) or object-verb-subject (OVS) structure, and grammatical function and thematic role relations were unambiguous for all critical trials. The speaker always inspected and mentioned the central character first, and, just after uttering the verb, she shifted her gaze to the post-verbal role filler (one of the outer two characters, the NP2 referent).

We analyzed fixations to the NP2 referent which started in the time window after the speaker gaze shift and before the NP2 referent was mentioned; which fell in that time window; as well as the onset latencies of the listeners’ first fixation to the NP2 referent after the speaker’s gaze shift. Together, these three measures provide insight into the time course with which listeners shift their attention toward the NP2 referent. If the speaker’s gaze rapidly affects listeners’ visual attention and language comprehension even in this non-frontal setting, then we should see faster post-verbal anticipation of the target character (the NP2 referent) when the speaker is visible (vs. when she is grayed out). If gaze-following is not robust in this setting, then perhaps only some listeners will be able to use it, leading to non-reliable effects of speaker gaze on participants’ visual anticipation of the post-verbal referent.

To test interactions of speaker gaze with sentence structure, we exploited a structural variation of German: both object- and subject-initial main clauses are grammatical, but the latter are canonical while the former are not. In reading, structurally unambiguous OVS sentences elicit longer reading times than SVO sentences, reflecting processing difficulty (e.g., Hemforth, 1993; Knoeferle and Crocker, 2009). During spoken comprehension, people can begin to anticipate the object referent of SVO sentences while hearing the verb (Knoeferle et al., 2005; Weber et al., 2006). For OVS sentences, by contrast, participants initially incorrectly anticipated an object- rather than a subject-NP2 referent at the verb, and this for both locally structurally ambiguous (Knoeferle et al., 2005; Weber et al., 2006) and unambiguous (Kamide et al., 2003) sentences. However, participants shifted their visual attention to the correct post-verbal (subject) referent when case marking and world knowledge (Kamide et al., 2003), intonation (Weber et al., 2006), or depicted events (Knoeferle et al., 2005) indicated the OVS structure of the sentence. If these context effects on syntactic structuring extend to speaker gaze, we should see later anticipation of the post-verbal (subject) referent for OVS sentences than of the post-verbal (object) referent for SVO sentences. Alternatively, if speaker gaze does not interact with syntactic structuring, we should see similar anticipation of the target referent for both SVO and OVS sentences after the speaker’s gaze shift. Finally, if speaker gaze combined with accusative (object) case marking can alleviate some of the difficulty of processing object-initial structures, we might see a numerically larger effect of speaker gaze on anticipation of the post-verbal referent for object- compared with subject-initial sentences.

To address the persistence of speaker gaze effects, and to ground the interpretation of the eye-movement pattern, we recorded response times and accuracy in a verification task that was substantially delayed after the speaker’s gaze shift and after sentence end. Faster RTs in this delayed task when the speaker is present (vs. absent), would corroborate the view that these effects can be long-lasting. In addition, if the systematic relationship between gaze-following and congruence effects (Staudte and Crocker, 2011) extends to our study, then we should see effects of gaze-following on response times also in our experiments, perhaps even in interaction with sentence structure (e.g., either SVO or OVS could benefit more from gaze-following). Alternatively, speaker gaze effects might be short-lived and not affect delayed, post-comprehension verification response times.

**Figure 1:**
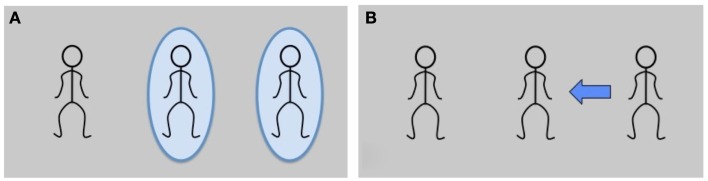
**Verification templates. (A)** Experiment 1, referent verification: Were the circled characters mentioned in the sentence? **(B)** Experiment 2, thematic role relations verification: Does the arrow reflect the thematic role relations of the sentence?

In Experiment 1, participants verified whether two depicted characters had (vs. had not) been mentioned in the sentence; this “referential” task served to replicate the results from prior studies (Hanna and Brennan, 2008; Staudte and Crocker, 2011). To ensure that any absence of interactions between speaker gaze and sentence structure in Experiment 1 were not the result of “shallow” processing for the referential task, Experiment 2 required participants to verify thematic role relations (see Figure 1).

## Experiments

### Participants

Participants for Experiment 1 (*N* = 32; 24 females; mean age = 22; *SD* = 2.8) and Experiment 2 (*N* = 32; 28 females; mean age = 24.3; *SD* = 4.3) were students at Bielefeld University and received 6 € for participating. All had normal or corrected-to-normal vision, were unaware of the purpose of the experiment, and signed an informed consent form.

### Materials and design

From a pool of 162 avatars in the virtual world “Second Life,” we selected 72 male characters that were identified unambiguously by 20 participants in a pilot naming test. These were combined into 24 triplets of characters and photographed in a neutral outdoor setting in Second Life. Twenty-four German subject-verb-object (SVO), and object-verb-subject (OVS) sentence pairs described a transitive action between the central character of a triplet (e.g., the waiter, “NP1 referent”) and one of the two outer characters (e.g., the millionaire, “NP2 referent”; see Table 1). The action itself was not depicted, so only the sentence identified the roles of agent and patient. From the sentence pairs and images we created 24 items consisting of four videos each: in the first two, the speaker could be seen producing either the SVO or the OVS version of the sentence [Table 1(a) and (b)] and looking at the characters in order of their mention (see Sentence Stimuli in Appendix for the sentence stimuli, and http://wwwhomes.uni-bielefeld.de/pknoeferle/Homepage/KnoeferleLab_Stimuli/MVI_10b_Kellner.MOV for an example video). The other two videos played back the identical SVO or OVS sentences, but the speaker was occluded [Table 1(c) and (d)]. Since the characters themselves did not move, this led to the impression of a static image with audio (which we will nonetheless refer to as a “video” in the following).

For the video recordings, a Canon PowerShot G10 camera was positioned approximately 1.5 m from the speaker. She was seated to the right of a 20″ Apple iMac 8.1 screen displaying the static scene with the three characters. In the recording, the speaker looked first at the camera and smiled. To give participants an example of what a gaze to each of the characters looked like, she next inspected them in a fixed order without speaking (middle, left, and right character). Before initiating the sentence, she shifted her gaze back to the middle character (the NP1 referent) and stayed there as she uttered the first noun phrase (mean speech onset: 6870 ms after video onset). Shortly after uttering the verb and before the second noun phrase, the speaker’s gaze shifted from the NP1 referent to the NP2 referent (shift onset: *M* = 949 ms after verb onset; *M* = 740 ms before NP2 onset). She looked back into the camera at the end of the sentence (total duration of the video: *M* = 13,143 ms).

For the post-video response task, we created verification templates with stick people as placeholders for the three avatar characters (see Figures 1A,B). In Experiment 1, the task was to verify whether both of the circled characters had been referenced by the sentence or not: for condition (c) in Table 1, the correct response to the template in Figure 1A would have been “yes,” since the positions of the waiter and the millionaire are circled and both were mentioned. In Experiment 2, participants verified whether the arrow on the template correctly (vs. incorrectly) characterized who-does-what-to-whom in the sentence. For Table 1(c) followed by Figure 1B, the response would be “no,” since the arrow points from the right character (the millionaire) to the waiter in the center. The waiter is not the patient of the sentence, but the agent, so a matching arrow would point from him outward, to the sentential patient (the millionaire). For experimental items, the matching arrow was always the reverse of the mismatching arrow (i.e., both matching and mismatching arrows connected the two mentioned characters); in filler trials, 50% of the arrows implicated the unmentioned character.

Overall, there were three within-subject factors: *speaker* (“gaze” vs. “no gaze”), *sentence structure* (SVO vs. OVS), and *congruence* of the sentence and the post-sentence template (congruous vs. incongruous). Only the first two factors were manipulated during the sentence and could therefore potentially affect online eye movements. Prior to the NP2, the case marking on the determiner of the NP1 (*Der* vs. *Den*) indicated constituent order (SVO and OVS, respectively), but not the identity of the NP2 referent. Since the nouns and the verb of all sentences were semantically and thematically unrelated, who-does-what-to-whom was never linguistically disambiguated prior to the second noun. By contrast, the speaker’s gaze shift to the NP2 referent could, in principle, prompt the listener to anticipate the NP2 referent. All three manipulated factors could affect post-sentence verification response times.

Counterbalancing ensured that half of the videos showed the NP2 referent on the right side of the screen, the other half on the left (i.e., the speaker shifted her gaze equally often to either side); it also ensured that the mentioned outer character in each video was equally often a thematic agent and patient; and that the “match” response was assigned to the left button on a button box for one half of the participants, and to the right button for the other half. Every participant experienced equal numbers of shifts to either side, as well as equal numbers of match/mismatch responses and of SVO/OVS sentences.

The three within-subject factors resulted in eight lists, which were presented in a different pseudo-randomized order for each participant. Each list contained one version of each of the 24 experimental items, and 48 filler items. These used a variety of sentence structures (e.g., subject-initial, dative-initial, passive, and prepositional constructions), combined with Second Life images or clipart depictions of action events. Half of the filler trials showed the speaker.

### Gaze-detection pretest

A gaze-detection pretest with a different group of participants (*N* = 20) examined how rapidly and accurately people could detect the speaker’s gaze shift from the NP1 to the NP2 referent. Participants watched the recorded videos and pressed a button as soon as they noticed that the speaker shifted her gaze away from the middle character after sentence start, indicating the direction of the shift. Detection accuracy was high (98%), and participants were fast to respond (*M* = 498 ms, *SD* = 386 ms). Note that the speaker moved both her head and her eyes to look at the relevant character, with the eyes shifting slightly before the onset of the head movement. This saccade was coded as the onset of the gaze shift and will be used in the analyses; however, we cannot exclude that it was possible for participants to make use of the head movement instead of the eye movement. Thus, the term “speaker gaze” does not refer to eye movements only; we use it in a wide sense to refer to the direction of attention by the speaker.

### Apparatus and procedure

Participants were seated in front of an Eyelink 1000 desktop head-stabilized eye tracker (SR Research) and the experimenter calibrated their right eye with a 9-point dot pattern. Participants were instructed on-screen that they would watch a series of unrelated videos which they should attend to and try to understand. They were informed that we were interested in the effect of different types of video complexity on memory retention, and were told that the main experiment would be followed by a memory test for the videos they had seen. This cover story was devised to mask the within-participant gaze manipulation and to ensure that participants paid attention to all aspects of the videos. They were further asked to verify as quickly and accurately as possible whether the post-video template matched (vs. did not match) the sentence.

Each participant completed four practice trials with feedback on their accuracy, followed by a second calibration; then the experiment began. Each trial started with a central fixation dot that participants fixated, followed by the video. As soon as this ended, the verification template appeared, and participants used a Cedrus response box to indicate whether the template matched the sentence (no feedback was provided during the experiment). Participants usually took a break half-way through the experiment, followed by recalibration; additional calibration was performed when necessary. The post-experiment memory test consisted of four practice and 24 experimental trials: participants inspected a snapshot from each of the 24 experimental (but not filler) videos in the same or the opposite speaker gaze condition as during the experiment. Their task in the memory test was to verify quickly and accurately whether these snapshots had (vs. had not) been present in the experiment. This resulted in a 2 × 2 design (*speaker*: gaze vs. no gaze; *previous occurrence*: yes vs. no). The experiment concluded with a debrief form and lasted 45–55 min.

## Analyses

### Response-time analyses

Response times (RTs) were time-locked to the display onset of the verification template. Using linear mixed models with crossed random intercepts and slopes for participants and items, we analyzed log-transformed RTs within 2 *SD* of each participant’s mean per congruence condition, including only trials with accurate responses. Details on model selection can be found in the Section “Model Selection Procedure for RTs and Eye-Movement Data”; the final models are listed in the Section “Details for the Linear Mixed Models Analyses” in the Appendix.

### Eye-movement analyses

Trials with recording problems (e.g., miscalibration, external noise, or track loss) and inaccurate responses were excluded from the analyses. Since we were most interested in the allocation of attention following the speaker’s gaze shift, we selected two primary time windows for the analyses (onsets and offsets for these were computed on a trial-by-trial basis): a “SHIFT” time window and an “NP2” time window. The SHIFT time window lasted for 800 ms from the onset of the speaker’s gaze shift in a particular video. In no gaze trials, the shift onset of the corresponding gaze video was used; this was possible because the underlying video was the same in both conditions and served to make the two maximally comparable. Across trials, the end-point of the SHIFT window corresponded roughly to the mean onset of the NP2 determiner (at *M* = 740 ms from shift onset; *SD* = 178). The NP2 window contained the following 800 ms, up to 1600 ms from shift onset, on a trial-by-trial basis. Roughly, this spanned the first half of the unfolding NP2 (NP2 offset: *M* = 1749 ms from shift onset, *SD* = 244). The two time windows were further split into 100 ms periods for some analyses, thus providing a detailed view of the time course with which speaker gaze affected fixations.

In both experiments and both time windows, we analyzed the mean log-gaze probability ratio with which listeners were likely to be fixating the target character over the competitor, and the target character over the NP1 referent. Additionally, we analyzed the log-transformed latencies of listeners’ first fixation to the target character after the speaker’s gaze shift, and the number of fixations to the target character in the SHIFT and NP2 time windows.

#### Log-gaze probability ratios

Mean log-gaze probability ratios were determined by dividing the probability of fixating the target character (aggregated over 20 ms bins) by the probability of fixating (a) the competitor [ln(*P*(target)*/P*(competitor))] or (b) the NP1 referent [ln(*P*(target)/*P*(NP1 ref))]^1^. A score of zero indicates that the two characters were fixated to an equal extent; a positive value implies that the target was fixated more than the competitor or the NP1 referent, and a negative value that it was fixated less. To analyze these probability ratios, we fitted separate linear models over participants and items^2^ (see Sections “Model Selection Procedure for RTs and Eye-Movement Data” and “Details for the Linear Mixed Models Analyses” in Appendix for details).

#### Onset latencies of the first target fixation after the speaker’s gaze shift

Fixation onset latencies were based on the first fixation to the target character made after the onset of the speaker’s gaze shift plus 100 ms. Such a post-shift fixation to the target character occurred in 99% of all accurate trials in Experiment 1, and 95% in Experiment 2. The onset of the speaker’s gaze shift was subtracted from the onset time of this fixation in order to obtain the latency in milliseconds from speaker gaze shift. We removed outliers ± 2 *SD* from each participant’s mean per gaze condition (Experiment 1: 24/739 trials; Experiment 2: 28/701) and log-transformed the data to reduce positive skew.

#### Model selection procedure for RTs and eye-movement data

Model selection followed the same procedure for analyses of response times, log-gaze probability ratios, and first fixation latencies. The initial model included two fixed factors for first fixation latencies (speaker and sentence structure), and three fixed factors each for response-time analyses (speaker, sentence structure, and congruence) and log probability ratios (speaker, sentence structure, and the 100 ms time windows^3^). All fixed factors were centered around a mean of zero to minimize collinearity, resulting in negative contrast coding (≈−0.5) for the factor levels no gaze, OVS, and incongruous, and positive contrast coding (≈ + 0.5) for gaze, SVO, and congruous, respectively. The eight levels of the time factor were also centered and ranged from ≈−3.5 to ≈+3.5. In addition, the initial models included all two-way interactions (and for RTs only: the three-way interaction of speaker, sentence structure, and congruence), as well as random intercepts for participants and/or items, and random slopes with all the fixed factors and their interactions. If this model did not converge, we removed interactions in the random parts of the model in rising order of variance explained, until convergence was achieved (note that the initial model always converged for log-gaze probability ratios).

The first converged model was defined as the “maximal model^4^,” against which subsequent simpler models were compared by log-likelihood ratio tests, following a backward selection procedure. We removed any fixed-effect interactions that did not contribute significantly to the maximal model, as well as their corresponding random slopes. This procedure continued until either the removal of a term led to a significant decrease in model fit (log-likelihood ratio), or until the model contained only main effects. The resulting model was designated our “final” model, for which we report the coefficients, *SE*, and *t*-values for all fixed effects and interactions (if present). Coefficients were considered significant if the absolute value of the *t*-statistic was greater than 2. Details on the final structure of all models can be found in Section “Details for the Linear Mixed Models Analyses” in Appendix.

#### Hierarchical log-linear analyses of fixation counts to the target

We also produced crosstables of fixation counts to the NP2 referent in the two time windows, for speaker × sentence structure^5^. The analyses were performed using backward elimination (see Field, 2005). For each time window, we performed one analysis with participant as random factor (1–32), and a second analysis with item as random factor (1–24). Reported partial χ^2^ and *p*-values are for the partial associations after inspection of *k*-way significance.

#### Relating real-time gaze-following to post-sentence gaze effects

To relate real-time gaze-following to post-sentence verification responses we performed two analyses. First, we analyzed correlations between eye movement and response-time measures: we determined for each fixation to the target character after the speaker’s gaze shift whether it occurred in the SHIFT time window, the NP2 time window, or thereafter, and then restructured the data to identify the first fixation in each trial to the target character (six trials without a fixation to the target character were excluded from Experiment 1, ten from Experiment 2). We computed difference scores by subtracting the no gaze count from the gaze count for each participant’s total number of trials with target fixations in the SHIFT period. Equivalent difference scores were created for first target fixation latency and RT from template onset. These difference scores were entered into correlation analyses.

Second, we entered a variable coding whether participants did or did not fixate the target character in the SHIFT time window into a linear mixed model of response times (log-transformed, outliers beyond 2 *SD* excluded, see the description of response-time analyses). The final model for Experiment 1 contained the centered factors congruence, the new factor *gaze-following*, and their interaction. In Experiment 2, the final model included sentence structure as well as congruence and gaze-following, and their interactions. We also included a random intercept and random slopes for all fixed factors by participants, and a random intercept only for items (the removal of random slopes was necessary to achieve convergence).

## Results for Experiment 1

### Accuracy and response-time results

In Experiment 1, one (bilingual) participant had to be replaced. Participants made at least 21/24 accurate responses in the verification task (>85%); their mean accuracy was 97%. Accuracy in the verification task was not modulated by the manipulated factors (χ^2^ tests: *p*s > 0.8). Accuracy in the post-experiment memory test was around chance (45%). Response times in the main experiment were significantly affected by congruence only: participants responded faster when the template was congruous (828 ms, *SD* = 309) than incongruous with the sentence (946 ms, *SD* = 30; coefficient = −0.07, *SE* > 0.01, *t* = −8.6; other *t*s < |1|).

### Eye-movement results

**Figure 2:**
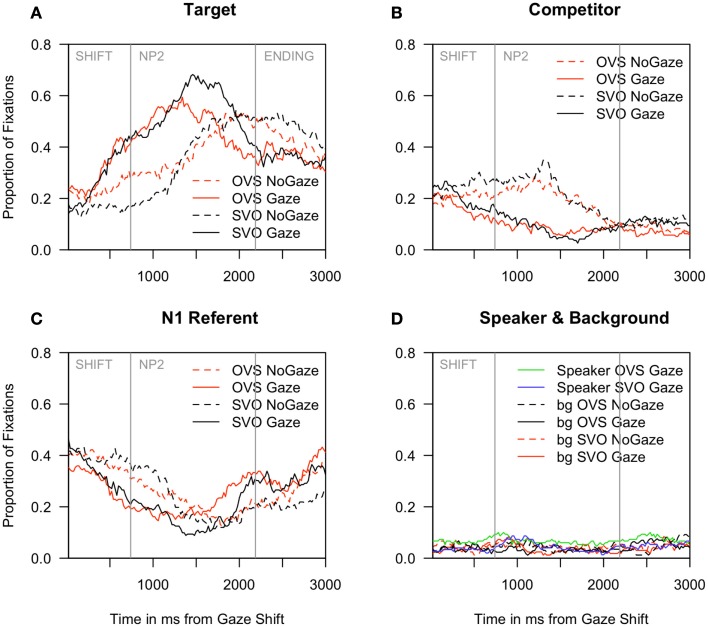
**Experiment 1: Proportion of fixations to (A) the target character, (B) the competitor, (C) the NP1 referent, and (D) the speaker region and the background**. All graphs begin at the onset of the speaker’s gaze shift. Mean onsets of the NP2 and the ending phrase are marked with vertical gray bars.

Inspection of eye-movement proportions in Figure 2A reveals that when the speaker was visible (gaze), fixations to the target character rose steeply 200 ms before it was mentioned (*M* = 740 ms from shift, *SD* = 178). By contrast, in cases where the speaker was not visible (no gaze), fixations to the target character increased only half-way through the NP2 that referenced it. Correspondingly, as can be seen in Figures 2B,C, fixations to the competitor and to the NP1 referent declined more quickly for the gaze than no gaze conditions. Although participants eagerly inspected the speaker before she began to speak, they hardly ever looked at her during the sentence: fixations to the speaker were as rare as to the background (Figure 2D).

#### Log-gaze probability ratio analyses

Table 2 presents the mean log-gaze probability ratios for the target vs. competitor and target vs. NP1 referent by condition for the SHIFT time window, and Table 3 the corresponding inferential analyses. Seeing the speaker inspect the target character increased listeners’ tendency to look at the target compared to the competitor; this gaze effect on target inspection increased over time and did not vary with sentence structure. In addition, participants were more likely to inspect the target over the competitor during OVS than SVO sentences. The NP1 referent was fixated more than the target in the no gaze condition, but less so in the gaze condition. It was also fixated more in SVO than in OVS sentences (the latter effect was reliable by participants only).

**Table 2:** **Experiment 1, SHIFT time window: Mean log-gaze probability ratios by condition for fixations to the target character (a) over the competitor or (b) over the NP1 referent**.

		Gaze	No gaze	Total
(a) Target vs.	SVO	0.39 (2.92)	−0.98 (2.65)	−0.3 (2.86)
competitor fixations	OVS	1.34 (2.68)	0.25 (2.76)	0.8 (2.77)
	Total	0.86 (2.84)	−0.36 (2.77)	0.25 (2.87)
(b) Target vs. N1	SVO	−0.27 (2.74)	−1.51 (3.02)	−0.89 (2.94)
referent fixations	OVS	0.38 (2.42)	−0.78 (2.68)	−0.20 (2.62)
	Total	0.05 (2.60)	−1.14 (2.87)	−0.55 (2.81)

*A positive number indicates preferred inspection of the target character; negative numbers preferred inspection of the competitor/NP1 referent. *SD* in parentheses*.

**Table 3:** **Experiment 1, SHIFT time window: Coefficients, *SE* and *t*-values for the final models of log-ratios of target fixations**.

	By participants			by items		
	Coefficient	*SE*	*t*-Value	Coefficient	*SE*	*t*-Value
**(A) TARGET VS. COMPETITOR**						
Intercept	0.25	0.20	1.25	0.30	0.17	1.83
Gaze	−1.22	0.33	**−3.71**	−1.24	0.31	**−3.95**
Structure	−1.09	0.33	**−3.51**	−0.81	0.36	**−2.26**
Time window	0.17	0.06	**2.94**	0.16	0.05	**3.48**
Gaze × structure	−0.28	0.06	−0.43	−0.52	0.69	−0.75
Gaze × time bin	−0.45	0.12	**−3.79**	−0.47	0.09	**−5.34**
Structure × time bin	<0.01	0.09	0.3	0.05	0.09	0.59
**(B) TARGET VS. N1 REFERENT**						
Intercept	−0.54	0.23	**−2.32**	−0.61	0.16	**−3.92**
Gaze	−1.20	0.39	**−3.11**	−1.15	0.30	**−3.85**
Structure	−0.69	0.27	**−2.59**	−0.46	0.27	−1.73
Time window	0.22	0.05	**4.63**	0.18	0.03	**5.18**
Gaze × structure	−0.09	0.63	−0.14	−0.57	0.45	−1.27
Gaze × time bin	−0.33	0.10	**−3.25**	−0.30	0.06	**−4.72**
Structure × time bin	0.03	0.06	0.44	<0.01	0.08	−0.04

*t-values in bold indicate a significant effect*.

In the NP2 time window, as participants heard the name of the NP2 referent, they were overall more likely to fixate the target than the competitor (significant intercept in Table 4; note that we have abstained from providing an overview of mean log-gaze probability ratios in the interest of readability, since the general pattern of results can be inferred from Figure 2). Crucially, participants fixated the target more when the speaker looked at it (*M* = 3.04, *SD* = 2.48), compared to when she was grayed out (*M* = 0.39, *SD* = 2.5), and this tendency continued to increase over the time window. Sentence structure no longer had any direct effect on log-gaze probability ratios, although it interacted with time bin by participants (greater increase in target fixations for SVO than OVS sentences).

**Table 4:** **Experiment 1, NP2 time window: Coefficients, *SE* and *t*-values for the final models of log-ratios of target fixations. The corresponding means and *SD* are included in the main text where necessary for interpretation**.

	By participants			By items		
	Coefficient	*SE*	*t*-Value	Coefficient	*SE*	*t*-Value
**TARGET VS. COMPETITOR FIXATIONS**						
Intercept	1.71	0.16	**10.95**	1.60	0.15	**10.86**
Gaze	−2.65	0.37	**−7.14**	−2.88	0.38	**−7.62**
Structure	−0.32	0.23	−1.39	−0.36	0.30	−1.22
Time bin	0.26	0.04	**6.31**	0.22	0.04	**5.59**
Gaze × structure	−0.25	0.64	−0.38	−0.41	0.75	−0.55
Gaze × time bin	−0.02	0.11	−0.22	0.01	0.08	0.15
Structure × time bin	0.21	0.08	**2.83**	0.09	0.11	0.89

*t-values in bold indicate a significant effect*.

#### Onset latency of first fixation to the NP2 referent

Participants began to fixate the target character earlier if they could see the speaker’s gaze shift (*M* = 832 ms from shift, *SD* = 562) than when they could not (*M* = 1165 ms, *SD* = 688). This speedup occurred for both sentence structures (mean differences for gaze vs. no gaze: 386 ms (SVO) and 282 ms (OVS); main effect of gaze: *t* = 4.81, coefficient = 0.35, *SE* = 0.07; main effect of structure: *t* < 1.5).

#### Hierarchical log-linear analyses of fixation counts

A reliable interaction of speaker and sentence structure in the SHIFT time window meant that while more anticipatory fixations to the target character occurred in the gaze condition than in the no gaze baseline, this increase was larger for SVO than OVS sentences [SVO: gaze 42% of all fixations vs. no gaze 17%; OVS: gaze 45% vs. no gaze 36%, see Figure 2; LRχ^2^(subj) = 9.80, *p* < 0.01; LRχ^2^(item) = 9.84, *p* < 0.01]. Overall, participants were more likely to anticipate the target when the speaker was visible vs. grayed out [LRχ^2^(subj/item) = 47.56, *p* < 0.001; variation as a function of participants], and in OVS vs. SVO sentences [LRχ^2^(subj/item) = 10.20, *p* = 0.001]. In the NP2 time window, the only significant effect in the partial associations was a main effect of speaker, with more looks to the target when the speaker was visible [gaze: 58% vs. no gaze: 41%, LRχ^2^(subj/item) = 17.97, *p* *<* 0.001].

#### Association between gaze-following and post-sentence effects

The difference scores for early fixation counts and first fixation latencies were highly correlated (Kendall’s *tau* = −0.69, *p* < 0.001), but neither was correlated with the response-time difference scores (both *p*s > 0.8). In the linear mixed model of response times, only congruence affected response times, as described above: participants reacted faster to congruous than incongruous templates (*t* = 7.7, coefficient = 0.16, *SE* = 0.02). Neither gaze-following nor its interaction with congruence significantly predicted response times (*t*s < 1).

## Summary and Discussion

Experiment 1 confirmed that participants anticipated the NP2 referent in NP1-V-NP2 sentences shortly after the speaker shifted gaze to that referent, and often even before its mention (see the hierarchical log-linear, log-gaze probability, and first fixation onset latency analyses). This seems to have been possible through peripheral vision, since the speaker was rarely fixated (see also Hanna and Brennan, 2008; Staudte and Crocker, 2011). It occurred in a setting in which the speaker was positioned at an angle to, rather than frontally opposite, the listener. Furthermore, the presence of the speaker interacted with sentence structure in affecting anticipatory shifts in attention to the target, but this interaction was found only in (hierarchical log-linear) analyses on fixations that *started* in the SHIFT time window.

In the post-sentence RTs, we found picture-sentence congruence effects (cf. Gough, 1965; Clark and Chase, 1972; Carpenter and Just, 1975; Staudte and Crocker, 2011), but speaker gaze and sentence structure effects were absent, and thus short-lived. Gaze-following during the sentence further did not correlate with post-sentence response times. The absence of speaker gaze effects on the RTs differs at first glance from the results in Staudte and Crocker (2011), who reported shorter response latencies for congruent vs. incongruent robot gaze. Unlike Staudte and Crocker (2011) who contrasted incongruent with congruent gaze, we contrasted congruent gaze with no gaze; this comparison may plausibly elicit less pronounced gaze effects. In addition, participants’ responses in their study were speeded and thus may have been more closely tied to incremental gaze effects during comprehension than our responses, which occurred much later: in our materials, the NP2 was followed by an unrelated end phrase (such as “outside the supermarket”), as well as by the verification template, so the average total time between the speaker’s gaze shift and the verification response was *M* = 14,068 ms (*SD* = 593 ms) – presumably ample time for any effects of gaze or structure to vanish.

One noteworthy point is that an interaction between speaker gaze and sentence structure in the SHIFT window emerged only in one out of three gaze measures. It is possible that sentence structure affects only some aspects of the eye-movement record. Alternatively, the task (verifying referents) encouraged participants to shy away from “deep” processing of sentence structure. Experiment 2 examined the latter possibility by changing the post-sentence task. Rather than verifying whether two circled characters had (vs. had not) been mentioned in the sentence, Experiment 2 used templates in which an arrow between two mentioned referents indicated who-does-what-to-whom, and was either congruous or incongruous with the thematic role relations of the sentence. Successful performance on this task requires computing the thematic role relations of the sentence and matching them against the depicted characters. To the extent that such a task focuses (visual) attention, an interaction of speaker gaze with sentence structure might be reflected in multiple eye-gaze measures and potentially even in post-sentence RTs. Experiment 2 thus provides a further opportunity to examine how seeing a speaker’s gaze shift interacts with syntactic structure building and incremental thematic role assignment.

## Results for Experiment 2

### Accuracy and response-time results

In Experiment 2, five participants had to be replaced (two bilingual; two misunderstood the task; one accuracy rate <75%). The remaining participants made at least 20/24 accurate responses; the mean accuracy of 96% did not vary by condition (*p*s > 0.85). Accuracy in the post-experiment memory task was around chance (52%).

Response times were significantly affected by both congruence and sentence structure: they were shorter when the template matched (vs. mismatched) the sentence (match: *M* = 969 ms, *SD* = 395; mismatch: *M* = 1149 ms, *SD* = 466; *t* = −6.07, coefficient: −0.17, *SE* = 0.03), and, unlike in Experiment 1, also shorter for SVO than OVS sentences (SVO: *M* = 1007 ms, *SD* = 347; OVS: *M* = 1114 ms, *SD* = 517; *t* = −2.39, coefficient: −0.05, *SE* = 0.02). The interaction of congruence and speaker approached significance (*t* = 1.79), with a greater difference in RTs (slower in the no gaze than gaze conditions) for incongruous compared to congruous trials.

### Eye-movement results

**Figure 3:**
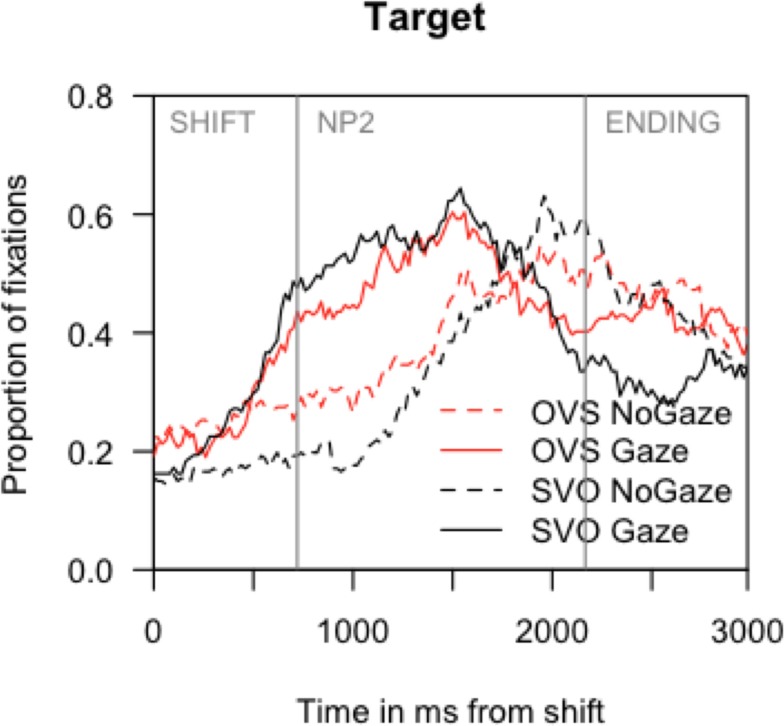
**Experiment 2: Proportion of fixations to the target character from the onset of the speaker’s gaze shift**. Mean onsets of the NP2 and the ending phrase are marked with vertical gray bars.

Figure 3 shows a steep increase of fixations to the target character during the SHIFT time window in conditions where the speaker was visible (gaze), just like in Experiment 1. By contrast, in the no gaze condition, fixations to the target character increased only in the second half of the NP2 window. Overall the gaze pattern was similar to that in Experiment 1; for the graphs of fixations to the other scene regions see Section “Detailed Graphs for Experiment 2” in Appendix.

#### Log-gaze probability ratio analyses

In the SHIFT window, participants fixated the target substantially more than the competitor or the NP1 referent when the speaker’s gaze was visible, and this tendency increased over time (see Tables 5 and 6). Unlike in Experiment 1, sentence structure had no consistent effect in either comparison, though in the by participants analysis it interacted with speaker gaze: in the no gaze condition there was a stronger preference for the NP1 referent over the target in SVO compared to OVS sentences, but this difference due to sentence structure was substantially reduced in the gaze condition.

**Table 5:** **Experiment 2, SHIFT time window: Mean log-gaze probability ratios by condition for fixations to the target character (a) over the competitor or (b) over the NP1 referent**.

		Gaze	No gaze	Total
(a) Target vs.	SVO	0.70 (2.67)	−0.54 (2.69)	0.08 (2.75)
competitor fixations	OVS	1.01 (2.99)	0.36 (2.72)	0.69 (2.87)
	Total	0.85 (2.83)	−0.09 (2.74)	0.38 (2.83)
(b) Target vs. N1	SVO	−0.16 (2.76)	−1.67 (2.61)	−0.92 (2.79)
referent fixations	OVS	−0.53 (2.63)	−0.57 (2.68)	−0.55 (2.65)
	Total	−0.35 (2.70)	−1.12 (2.70)	−0.73 (2.72)

**SD* in parentheses*.

**Table 6:** **Experiment 2, SHIFT time window: Coefficients, *SE* and *t*-values for the final models of log-ratios of target fixations**.

	By participants			By items		
	Estimate	*SE*	*t*-Value	Estimate	*SE*	*t*-Value
**(A) TARGET VS. COMPETITOR FIXATIONS**						
Intercept	0.38	0.17	**2.27**	0.34	0.21	1.65
Gaze	−0.94	0.36	**−2.63**	−1.23	0.29	**−4.17**
Structure	−0.60	0.38	−1.58	−0.55	0.35	−1.55
Time bin	0.23	0.05	**4.57**	0.22	0.05	**4.17**
Gaze × structure	−0.59	0.64	−0.92	−0.33	0.73	−0.45
Gaze × time bin	−0.37	0.10	**−3.57**	−0.42	0.09	**−4.82**
Structure × time bin	0.16	0.09	1.85	0.24	0.11	**2.23**
**(B) TARGET VS. N1 REFERENT FIXATIONS**						
Intercept	−0.73	0.25	**−2.89**	−0.84	0.18	**−4.79**
Gaze	−0.77	0.26	**−3.03**	−0.87	0.25	**−3.46**
Structure	−0.37	0.32	−1.13	−0.39	0.27	−1.45
Time bin	0.24	0.04	**6.69**	0.22	0.03	**6.32**
Gaze × structure	1.48	0.63	**−2.34**	−0.65	0.58	−1.12
Gaze × time bin	−0.39	0.08	**−5.12**	−0.28	0.05	**−5.04**
Structure × time bin	0.02	0.08	0.28	0.12	0.08	1.45

*t-values in bold indicate a significant effect*.

The pattern of results in the NP2 time window largely matched the earlier time window: participants fixated the target substantially more than the competitor in the gaze than no gaze conditions (*M* = 3.61, *SD* = 2.23 vs. *M* = 0.40, *SD* = 2.62), and this tendency continued to increase (see Table 7 for the inferential analyses). As in the SHIFT window, sentence structure interacted with speaker gaze in the by participants analysis (a greater difference between no gaze and gaze for SVO than OVS sentences).

**Table 7:** **Experiment 2, NP2 time window: coefficients, *SE* and *t*-values for the final models of log-ratios of target fixations**.

	By participants			By items		
	Estimate	*SE*	*t*-Value	Estimate	*SE*	*t*-Value
**TARGET VS. COMPETITOR FIXATIONS**						
Intercept	1.78	0.18	**10.01**	1.78	0.13	**13.50**
Gaze	−2.76	0.37	**−7.41**	−2.73	0.27	**−10.05**
Structure	−0.29	0.34	−0.86	−0.36	0.39	−0.92
Time Bin	0.12	0.04	**2.81**	0.12	0.05	**2.31**
Gaze × structure	−1.10	0.50	**−2.18**	−0.07	0.61	−0.11
Gaze × time bin	0.08	0.09	0.88	0.11	0.09	1.25
Structure × time bin	−0.06	0.08	−0.77	−0.05	0.08	−0.71

*Descriptive statistics are included in the main text*.

*t-values in bold indicate a significant effect*.

#### Onset latency of first fixation to the NP2 referent

Again, participants were faster to fixate the target character when they could (vs. could not) see the speaker’s gaze shift. Unlike in Experiment 1 however, sentence structure also interacted with speaker gaze: in SVO sentences, participants fixated the target character 358 ms earlier with gaze than with no gaze. This difference was substantially smaller for OVS sentences (*M* = 155 ms; *t* = 2.73, coefficient: 0.29, *SE* = 0.11).

#### Hierarchical log-linear analyses of fixation counts

In the SHIFT time window, hierarchical log-linear analyses confirmed a main effect of speaker [gaze: 39% vs. no gaze: 28% of target fixations, LRχ^2^(subj/item) = 13.07, *p* < 0.001]. As in the other two analyses, speaker gaze and sentence structure interacted by participants [LRχ^2^(subj) = 8.04; LRχ^2^(item) = 7.48, *p*s < 0.01], with a substantially larger effect of speaker for SVO (17%) than for OVS sentences (6%).

#### Association between gaze-following and post-sentence effects

**Figure 4:**
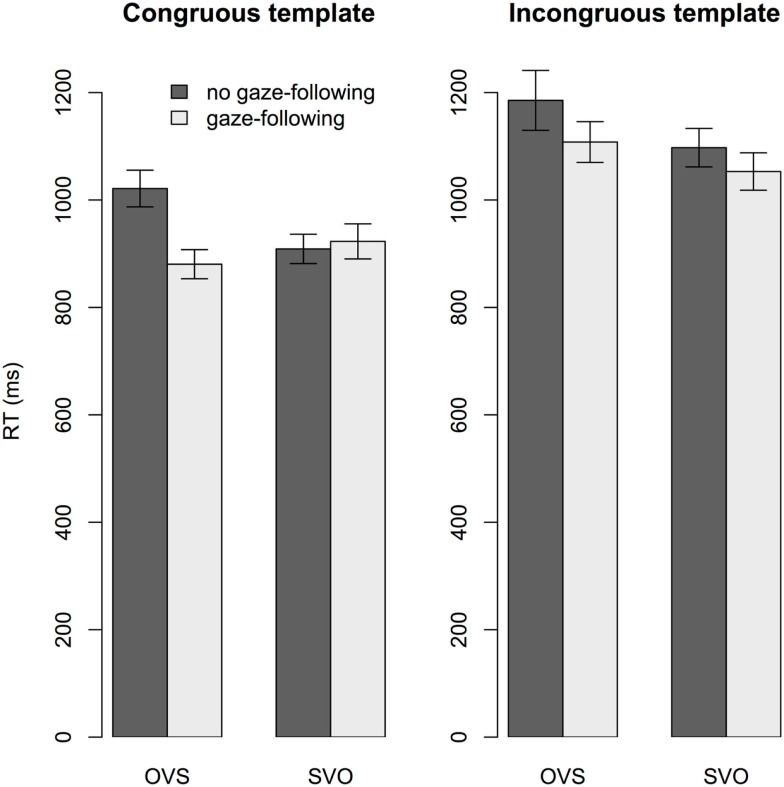
**Experiment 2: Interaction effect of gaze-following, sentence structure and congruence on response times**. Since we collapsed across the factor speaker, the bars labeled “gaze-following” include both gaze and no gaze trials, whenever the target character was fixated in the SHIFT time window. In these trials, participants’ response times to OVS sentences were shorter than for OVS sentences when they did not follow gaze, but only when the template matched the sentence. Error bars are *SE*.

The difference scores for early fixation counts and first fixation latencies were highly correlated (Kendall’s *tau* = −0.66, *p* < 0.001), but neither correlated with the response-time difference scores (*p*s > 0.5). The final model of response times revealed a reliable effect of both sentence structure (*t* = −2.33, coefficient = −0.05, *SE* = 0.02) and gaze-following (*t* = −2.46, coefficient = −0.05, *SE* = 0.02): Participants were faster to respond for SVO than OVS sentences (*M* = 997 ms vs. *M* = 1047 ms), and faster when they had (vs. had not) followed the speaker’s gaze (*M* = 994 ms vs. *M* = 1045 ms, respectively). Figure 4 clarifies the reliable three-way interaction (*t* = −2.52, coefficient = −0.18, *SE* = 0.07): The facilitatory effect of gaze-following on response times was more pronounced for OVS than SVO sentences, but only when the template matched the sentence.

## Summary and Discussion

The results from Experiment 2 replicated the rapid speaker gaze effects on listeners’ sentence comprehension and visual attention to the target. In all three eye-movement analyses, the speaker gaze effect was larger for SVO than OVS sentences in the SHIFT window. This interaction of speaker gaze and sentence structure continued into the NP2 time window, though it was reliable by participants only (log-gaze probability ratios).

Unlike in Experiment 1, responses were faster when participants had vs. had not followed the speaker’s gaze, and for SVO than OVS sentences. Gaze-following eliminated the SVO-OVS difference, but only when the template matched the sentence (see Figure 4). Thus, following a speaker’s gaze during sentence comprehension in preparation for verifying thematic role relations alleviated the difficulty involved in understanding OVS sentences.

Overall, the task focus on thematic role relations verification brought out interactions of speaker gaze and sentence structure in both time windows and in all three measures. This highlights the important influence of the listener’s current comprehension goal on online visual attention (see Salverda et al., 2011). Moreover, it provides strong evidence for the processing of speaker gaze in close temporal coordination with incremental syntactic structuring and thematic interpretation.

## General Discussion

The present research examined three issues regarding effects of speaker gaze on a comprehender’s visual attention and language comprehension. We asked (a) whether speaker gaze effects extend from a frontal speaker-listener setup to settings where the speaker is positioned at an angle relative to the comprehender (which arguably makes gaze shifts harder to detect); (b) whether speaker gaze merely enables the anticipation of referents, or whether it is also linked to other comprehension processes such as syntactic structuring and incremental thematic role assignment; and (c) whether speaker gaze effects on a listener’s visual attention are short-lived or last into substantially delayed verification processes after sentence end. We recorded participants’ eye movements as they inspected videos of a speaker who shifted her gaze to the NP2 referent of a subject-verb-object (SVO) or object-verb-subject (OVS) sentence shortly after producing the verb. We also recorded participants’ response latencies in a delayed, post-sentence verification task on whether two characters had (vs. had not) been mentioned in the sentence (Experiment 1) or whether depicted thematic role-relations matched (vs. did not match) the sentential thematic role relations (Experiment 2). From this investigation, we gained insight into the extent to which speaker gaze effects play a role during real-time language comprehension and should thus be accommodated by existing accounts of situated language comprehension (e.g., Knoeferle and Crocker, 2006, 2007; Altmann and Kamide, 2009).

In both the reference- and the role-relations verification tasks, listeners’ eye gaze rapidly followed the speaker’s gaze shift to the NP2 referent before the speaker mentioned it. Thus, a speaker’s gaze shift to an upcoming referent can elicit rapid shifts in the visual attention of listeners – not only in a frontal speaker-listener setting (Hanna and Brennan, 2008; Staudte and Crocker, 2011), but also when the speaker is angled by 45–60° relative to the listener.

Speaker gaze moreover rapidly affected core comprehension processes such as syntactic structuring and thematic role assignment, on top of referential anticipation. The syntactic structure of the sentence clearly modulated listeners’ visual anticipation of the NP2 referent when the task focused the listener’s attention on processes of thematic role interpretation (Experiment 2). In fact, this effect was observed in one of the gaze measures even when the task was referent verification and thus did not require “deep” processing of the syntactic structure and thematic role relations (Experiment 1).

Participants made earlier first fixations to the target and generally fixated it more when they saw the speaker shift her gaze to this character, a gaze benefit that was more pronounced (faster and a greater numerical difference) for SVO than OVS sentences. Moreover, speaker gaze effects continued well into the NP2, and even extended to responses that were made considerably later, at least when participants verified the thematic role relations of the sentence. In fact, gaze and sentence structure interacted in modulating the response times: SVO sentences were verified faster overall, but when participants followed gaze during OVS sentences in the congruous condition, their mean response times were as fast as for SVO sentences. Thus, gaze-following can eliminate the difficulty associated with the processing of OVS sentences.

The cross-situational robustness of speaker gaze effects and their interaction with syntactic structuring and thematic role assignment suggest that existing accounts of visually situated language comprehension should accommodate them. The Coordinated Interplay Account predicts visual context effects closely temporally coordinated with when relevant aspects of visual context are identified by language. In line with this prediction, the present findings contribute the insight that the listener’s gaze shift to the target character occurred in close temporal coordination with the speaker’s gaze shift (see also, e.g., Hanna and Brennan, 2008; Staudte and Crocker, 2011). Likewise, evidence for interactions of speaker gaze effects with sentence structure appeared shortly after the speaker’s gaze shift. Overall, the reported results fit with the prediction that visual context effects will appear closely time-locked to when visual context information is identified as relevant; by contrast, the accounts do not yet accommodate the outcome of verification processes (but see Knoeferle et al., in preparation).

With regard to the mechanism through which speaker gaze informs language comprehension, its effects likely differ compared to depicted referents or actions, which have been the focus of attention in previous studies. For depicted actions, for instance, a referential “match” with the verb can clarify that an action is relevant for comprehension. By contrast, speaker gaze is neither referenced nor associated with lexical entries, so its relevance at a given point in time must be computed differently. This could happen via knowledge of the functional role of the speaker in the communicative process, together with peripherally perceived dynamic motion (e.g., of gaze and head shifts).

Overall, while a direct comparison of action and speaker gaze effects will determine the extent of their similarities, the present findings clarify that speaker gaze effects are robust to variation in speaker-listener position; that they not only enable referential anticipation but also interact with core comprehension processes such as syntactic structuring and thematic role assignment; and that there are situations in which they extend in time and scope beyond the end of the current sentence to influence response times in a delayed verification task.

## Conflict of Interest Statement

The authors declare that the research was conducted in the absence of any commercial or financial relationships that could be construed as a potential conflict of interest.

## Footnotes

^1^Since log-ratios are undefined for 0, we replaced counts of 0 in numerator or denominator by 0.1 before the division.

^2^Since the log ratio measure relies on aggregation, it is not possible to include crossed random effects of participants and items in the same linear mixed model.

^3^We also looked at models without the time factor: these found comparable results, but with a worse fit.

^4^We always compared our maximal models to models with the same fixed effects structure but no random slopes (random intercepts only). The pattern of results was the same, but model fit suffered.

^5^Initial tables also contained the other scene regions (speaker, NP1 referent, competitor character, and background), but this approach had to be abandoned due to sparse frequency counts in many cells. Sufficient expected counts are a requirement of hierarchical log-linear analyses.

## Appendix

### Sentence stimuli

The following list contains the sentence stimuli first in subject-verb-object, then in object-verb-subject sentence structure. The same ending phrase was used in both conditions. The videos can be obtained from the authors upon request.

(1) Der Arzt verhext den Bettler…/Den Arzt verhext der Taucher vor dem Frühstück.
(2) Der Biker bedient den Harlekin…/Den Biker bedient der Dirigent neben der Tankstelle.
(3) Der Tennisspieler bespitzelt den Wikinger…/Den Tennisspieler bespitzelt der Chinese in der Pause.
(4) Der Fußballer belohnt den Radfahrer…/Den Fußballer belohnt der Schuljunge des Öfteren.
(5) Der Gärtner verprügelt den Feuerwehrmann…/Den Gärtner verprügelt der Basketballer nach der Arbeit.
(6) Der Geschäftsmann kitzelt den Bogenschützen…/Den Geschäftsmann kitzelt der Baseballspieler nahe der Kirche.
(7) Der Holländer verhaftet den Bauarbeiter…/Den Holländer verhaftet der Indianer ohne jede Scheu.
(8) Der Skifahrer frisiert den Wanderer…/Den Skifahrer frisiert der Zauberer im Grünen.
(9) Der Fotograf ersticht den Butler…/Den Fotografen ersticht der Schotte in den Ferien.
(10) Der Kellner beglückwünscht den Millionär…/Den Kellner beglückwünscht der Saxofonist außerhalb des Geschäfts.
(11) Der Volleyballer attackiert den Mönch…/Den Volleyballer attackiert der Clown zur Mittagszeit.
(12) Der Chemiker interviewt den Hooligan…/Den Chemiker interviewt der Ruderer im Park.
(13) Der Maler grüßt den Detektiv…/Den Maler grüßt der Holzfäller bei jeder Gelegenheit.
(14) Der Lehrer fotografiert den Rapper…/Den Lehrer fotografiert der Angler unweit der Stadt.
(15) Der Mechaniker verfolgt den Piraten…/Den Mechaniker verfolgt der Hofnarr trotz aller Gefahr.
(16) Der Neandertaler bestraft den Fechter…/Den Neandertaler bestraft der Opa voller Überzeugung.
(17) Der Opernsänger überfällt den Jäger…/Den Opernsänger überfällt der Ritter vollkommen überraschend.
(18) Der Polizist beschenkt den Kameramann…/Den Polizisten beschenkt der Gewichtheber abseits des Feldes.
(19) Der Skater vergiftet den Cowboy…/Den Skater vergiftet der Golfer am Nachmittag.
(20) Der Punk bekocht den Zeitungsjungen…/Den Punk bekocht der Mafiaboss nächsten Winter.
(21) Der Reiter erschießt den Schornsteinfeger…/Den Reiter erschießt der Zauberkünstler vor der Abreise.
(22) Der Grieche kostümiert den Astronauten…/Den Griechen kostümiert der Gitarrist im Garten.
(23) Der Rollstuhlfahrer verwarnt den Pfadfinder…/Den Rollstuhlfahrer verwarnt der Franzose wenig freundlich.
(24) Der Schlittschuhläufer bewirtet den Priester…/Den Schlittschuhläufer bewirtet der Surfer am Morgen.

### Details for the linear mixed models analyses

Overview of the final models across experiments and analyses. The factors “match,” “gazefollow,” “gaze,” and “struc” are place holders for the experimental variables congruency, gaze-following, speaker, and sentence structure, respectively. These predictors were centered around 0, so that the factor levels incongruous, no gaze-following, no gaze, and OVS were negatively coded, while congruous, gaze-following, gaze, and SVO were positive. With regard to log-gaze probability ratios, the same final models were obtained both for the comparisons of target over competitor vs. target over NP1 referent fixations, and for the SHIFT and NP2 time windows. The *R*^2^ and sigma values are approximated in these cases.

**Table A1:**

		Final model structure	*R*^2^	Sigma
**EXPERIMENT 1: REFERENCE VERIFICATION**				
Response times		logrt ∼ match + gaze + struc + (1 + match + gaze + struc | participant) + (1 + item)	0.61	0.08
		logrt ∼ match*gazefollow + (1 + match*gazefollow | participant) + (1 + item)	0.60	0.19
Log-gaze probability ratio	Participants	lograt ∼ gaze*struc + gaze*time + struc*time + (1 + gaze*struc + gaze*time + struc*time + participant)	>0.7	<1.5
	items	lograt ∼ gaze*struc + gaze*time + struc*time + (1 + gaze*struc + gaze*time + struc*time + item)	>0.7	<1.3
First fixation latency		logfixlat ∼ gaze + struc + (1 + gaze + struc | participant) + (1 + gaze + struc | item)	0.30	0.63
**EXPERIMENT 2: ROLE RELATIONS VERIFICATION**				
Response times		logrt ∼ match*gaze + struc + (1 + match + gaze + struc | participant) + (1 | item)	0.57	0.21
		logrt ∼ match*gazefollow*struc + (1 + gazefollow + match*struc ∼ participant) + (1 ∼ item)	0.56	0.21
Log-gaze probability ratio	Participants	lograt ∼ gaze*struc + gaze*time + struc*time + (1 + gaze*struc + gaze*time + struc*time | participant)	>0.7	<1.5
	items	lograt ∼ gaze*struc + gaze*time + struc*time + (1 + gaze*struc + gaze*time + struc*time | item)	>0.7	<1.3
First fixation latency		logfixlat ∼ gaze*struc + (1 + gaze*struc | participant) + (1 + gaze + struc | item)	0.24	0.69

### Detailed graphs for experiment 2

#### Experiment 2

Proportion of fixations to (a) the competitor and (b) the NP1 referent. Graphs begin at the onset of the speaker’s gaze shift. Mean onsets of the NP2 and the ending phrase are marked with vertical gray bars.

## References

[B1] Altmann G. T. M. (2004). Language-mediated eye-movements in the absence of a visual world: the ‘blank screen paradigm’. Cognition 93, B79–B87 10.1016/j.cognition.2004.02.005 15147941

[B2] Altmann G. T. M. Kamide Y. (2007). The real-time mediation of visual attention by language and world knowledge: linking anticipatory (and other) eye movements to linguistic processing. J. Mem. Lang. 57, 502–518 10.1016/j.jml.2006.12.004

[B3] Altmann G. T. M. Kamide Y. (2009). Discourse-mediation of the mapping between language and the visual world: eye movements and mental representation. Cognition 111, 55–71 10.1016/j.cognition.2008.12.005 19193366 PMC2669403

[B4] Campana E. Silverman L. Tanenhaus M. K. Bennetto L. Packard S. (2005). Real-Time Integration of Gesture and Speech During Reference Resolution, pp. 378–383 Mahwah, NJ: Lawrence Erlbaum

[B5] Carpenter P. A. Just M. A. (1975). Sentence comprehension: a psycholinguistic processing model of verification. Psychol. Rev. 82, 45–73 10.1037/h0076248

[B6] Chambers C. G. Tanenhaus M. K. Magnuson J. S. (2004). Actions and affordances in syntactic ambiguity resolution. J. Exp. Psychol. Learn. Mem. Cogn. 30, 687–696 10.1037/0278-7393.30.3.687 15099136

[B7] Clark H. H. Chase W. G. (1972). On the process of comparing sentences against pictures. Cogn. Psychol. 3, 472–517 10.1016/0010-0285(72)90019-9

[B8] Cline M. (1967). The perception of where a person is looking. Am. J. Psychol. 80, 41–50 10.2307/1420539 6036357

[B9] Dahan D. Tanenhaus M. (2005). Looking at the rope when looking for the snake: conceptually mediated eye movements during spoken-word recognition. Psychon. Bull. Rev. 12, 453–459 10.3758/BF03193787 16235628

[B10] Field A. (2005). Introduction to Statistics Using SPSS. London: Sage Publications

[B11] Gibson J. Pick A. (1963). Perception of another person’s looking behavior. Am. J. Psychol. 76, 386–394 10.2307/1419779 13947729

[B12] Gough P. B. (1965). Grammatical transformations and speed of understanding. J. Verbal Learning Verbal Behav. 5, 107–111 10.1016/S0022-5371(65)80093-7

[B13] Hanna J. Brennan S. (2008). Speaker’s eye gaze disambiguates referring expressions early during face-to-face conversation. JML 57, 596–615

[B14] Hemforth B. (1993). Kognitives Parsing: Repräsentation und Verarbeitung sprachlichen Wissens. Sankt Augustin: Infix-Verlag

[B15] Holle H. Obermeier C. Schmidt-Kassow M. Friederici A. D. Ward J. Gunter T. C. (2012). Gesture facilitates the syntactic analysis of speech. Front. Psychol. 3:74 10.3389/fpsyg.2012.00074 22457657 PMC3307377

[B16] Huettig F. Altmann G. T. M. (2004). “The online processing of ambiguous and unambiguous words in context: evidence from head-mounted eye-tracking,” in The On-Line Study of Sentence Comprehension: Eyetracking, ERP and Beyond, eds Carreiras M. Clifton C. (New York: Psychology Press), 187–207

[B17] Kamide Y. Scheepers C. Altmann G. T. M. (2003). Integration of syntactic and semantic information in predictive processing: cross-linguistic evidence from German and English. J. Psycholinguist. Res. 32, 37–55 10.1023/A:1021933015362 12647562

[B18] Knoeferle P. Carminati M. N. Abashidze D. Essig K. (2011). Preferential inspection of recent real-world events over future events: evidence from eye tracking during spoken sentence comprehension. Front. Cogn. (special issue edited by Myachykov A. Scheepers C. Shtyrov Y.) 2:376 10.3389/fpsyg.2011.00376 PMC3245670 22207858

[B19] Knoeferle P. Crocker M. W. (2006). The coordinated interplay of scene, utterance, and world knowledge: evidence from eye tracking. Cogn. Sci. 30, 481–529 10.1207/s15516709cog0000_65 21702823

[B20] Knoeferle P. Crocker M. W. (2007). The influence of recent scene events on spoken comprehension: evidence from eye-movements. J. Mem. Lang. 75, 519–543 10.1016/j.jml.2007.01.003

[B21] Knoeferle P. Crocker M. W. (2009). Constituent order and semantic parallelism in on-line comprehension: eye-tracking evidence from German. Q. J. Exp. Psychol. 62, 2338–2371 10.1080/17470210902790070 19418379

[B22] Knoeferle P. Crocker M. W. Scheepers C. Pickering M. J. (2005). The influence of the immediate visual context on incremental thematic role-assignment: evidence from eye-movements in depicted events. Cognition 95, 95–127 10.1016/j.cognition.2004.03.002 15629475

[B23] Richardson D. C. Dale R. (2005). Looking to understand: the coupling between speakers and listeners eye movements and its relationship to discourse comprehension. Cogn. Sci. 29, 1045–1060 10.1207/s15516709cog0000_29 21702802

[B24] Salverda A. P. Brown M. Tanenhaus M. K. (2011). A goal-based perspective on eye movements in visual-world studies. Acta Psychol. 137, 172–180 10.1016/j.actpsy.2010.09.010 PMC3109199 21067708

[B25] Sedivy J. C. Tanenhaus M. K. Chambers C. G. Carlson G. N. (1999). Achieving incremental semantic interpretation through contextual representation. Cognition 71, 109–148 10.1016/S0010-0277(99)00025-6 10444906

[B26] Staudte M. Crocker M. W. (2011). Investigating joint attention mechanisms through spoken human–robot interaction. Cognition 120, 268–291 10.1016/j.cognition.2011.05.005 21665198

[B27] Tanenhaus M. K. Spivey-Knowlton M. J. Eberhard K. Sedivy J. C. (1995). Integration of visual and linguistic information in spoken language comprehension. Science 268, 632–634 10.1126/science.7732367 7777863

[B28] Weber A. Grice M. Crocker M. W. (2006). The role of prosody in the interpretation of structural ambiguities: a study of anticipatory eye movements. Cognition 99, B63–B72 10.1016/j.cognition.2005.07.001 16157327

[B29] Wu Y. C. Coulson S. (2005). Meaningful gestures: electrophysiological indices of iconic gesture comprehension. Psychophysiology 42, 654–667 10.1111/j.1469-8986.2005.00356.x 16364061

